# The Influence of High Air-Temperatures

**Published:** 1905-11-18

**Authors:** 


### Hospital Clinics.

### THE INFLUENCE OF HIGH AIR-TEMPERATURES.

Dr. J. S. Haldane has published in the current issue of *The Journal of Hygiene* a most interesting and valuable study of this subject. The object of the investigation was to determine the limits within which high air-temperatures can be tolerated without evidence of essential disturbance to the human economy, and also to ascertain the direction of departures from the normal when the limit of tolerance is passed. The subject, as the author observes, is of the widest application, including, as it does, the influence of warm weather and of tropical heat, as well as the problems attached to certain industrial occupations, the followers of which are constantly being exposed to great heat. This latter class is a very large one, embracing miners, the engine-room and stokehold complements of ships, those engaged in the drawing of ovens in the pottery trade, and many others.

It was long ago discovered that the tolerance of high temperatures was mainly a matter of evaporation of moisture from the surface of the body, that is to say that in the presence of a rapid evaporation of the surface moisture a high degree of heat-tolerance is the rule; but if the hot air is heavily charged with aqueous vapour, tolerance of the heat it carries is much reduced. This doctrine, of course, chimes with the experience of all who live in tropical climates—namely, that moist heat is a source of far greater discomfort than is dry heat. Experiments made by Blagden Forsyth and Dobson in the year 1775 showed that these observers could bear for a few minutes an air-temperature of 250° F. without serious inconvenience, though a beef-steak exposed to the same heat in the same place was cooked in thirteen minutes.

A preliminary investigation was conducted by Dr. Haldane and his assistants in order to determine the normal limits of variation in the body-heat during ordinary good health, the results of which are of very considerable intrinsic interest.

Firstly, with regard to the temperature in the rectum, which is undoubtedly the most reliable index of the deep body-temperature. Pembrey and Nichol consider that the difference between the maximum and minimum temperatures observed in the rectum during health is a range of about 2.2° F., that is to say, from 99.4° to 97.2°. But the author states positively that muscular effort or hot weather will enlarge this variation considerably, for after a walk lasting one hour a rectal temperature of 101.3° F. has been noted in a healthy individual. Further, in the case of eighty-three soldiers, the temperature of whose urine was examined on a warm afternoon when the air-temperature was 88.5°, the average of all the temperatures was 99.6° F., the maximium being 100.8° F. These men were all in apparently robust health, they were lightly clad, and had not recently undergone any physical labour. In the author's opinion the maximum limit of rectal temperature in health ought not to be placed lower than 101° if the surrounding air be warm and if the subject of the experiment has been engaged in muscular labour; under contrary circumstances, of course—that is to say idleness and cold weather—such a temperature in the rectum would not be natural.

Oral temperatures are very deceptive, especially in cold weather and after hard physical exertion: the proximity of the skin surface and of the air-passages tends to reduce the temperature of the mouth considerably below that of the body generally; a difference of 4.5° F. has been observed between the rectal and oral temperatures in the same subject at the same time. It is clear, therefore, that no scientific accuracy is to be expected from mouth-temperatures. The ratio borne by the temperature of the mouth to that in the rectum varies, besides, in each individual, a fact which precludes deduction of the real body heat from a consideration of the mouth-temperature.

Some of the observations forming the basis of the communication under notice were carried out in the Levant and Dolcoath mines, and indicate, therefore, the conditions under which a section of the community passes a large portion of life. The Levant mine is a tin and copper mine running out for a mile or so under the Atlantic Ocean from the coast of Cornwall. Not unnaturally under the circumstances the ventilation of the mine is very inadequate, and its air-temperature high owing to the heat developed by the oxidation of iron pyrites. The air is saturated with moisture, and the temperature ranges in different levels from 80° to 93° F. On the occasion of his first visit to this mine Dr. Haldane experienced great discomfort; with an air-temperature about him which did not exceed 86° F., his mouth-temperature gradually rose to 105.2° F., while another novice in the party showed a mouth-temperature of only 101.5° F., and that after deliberate walking only.

A series of important experiments were carried out in the Dolcoath mine, of which series the following may be taken as a type. The subject of the experiment entered the mine with a rectal temperature of 100.2° F. and a mouth-temperature of 99.2. He took up his position in a hot level where the surrounding air-temperature was 94° F. as shown by the dry-bulb and 93.6° F. as shown by the wet-bulb thermometer; that is to say the air about him was saturated with aqueous vapour. He was lightly clad and doing nothing, yet in thirteen minutes he was sweating profusely. In an hour he was conscious of a throbbing in his head, while his pulse-rate had risen to 138. In a little over two hours his rectal temperature had actually reached 104.2° F., while his mouth-temperature was 102.1° F., and breathing was becoming progressively more laborious. He left the level at this stage, and within half an hour was completely recovered.

The indication offered by this experiment is that the subject of it was incapable of maintaining a normal body-temperature in still and saturated air at a temperature of 94° F. A subsequent trial showed that under such conditions of stillness and saturation, air whose heat did not exceed 89° F. was still hot enough to upset the automatic regulation of temperature in the body. Another point of interest was that the temperature, having once begun to rise, did not rapidly reach an equilibrium proportional to the excess of heat in the surroundings, but continued to rise steadily and was still rising after two and three quarter hours.

A large number of experiments were carried out under experimental conditions to indicate the bearing of exercise, and of movement in the air, upon the tolerance of high-temperatures. These we cannot here detail, although they will be found full of interest for students of industrial hygiene. The essential conclusions to which they point are, however, as follows:—

The rectal temperature in a human subject exposed to hot air does not advance abnormally until the air-temperature, as measured by the wet-bulb thermometer, has reached the neighbourhood of 88° F. This, at least, is the case assuming that the air is still, and that the subject is either stripped to the waist or clad in light flannel. But if the air-temperature exceeds this limit by even one degree there follows a marked rise in the rectal temperature, which rise is continuous, and shows no inclination to cease so long as the subject remains under the conditions which originated it. This rise of temperature becomes progressively more rapid in proportion as the critical temperature (88° F.) is left further behind. That is to say that for a wet-bulb air temperature of 89° or 90° F., the rise of rectal temperature per hour is from 1° to 1.4° F., while for an air-temperature of 98° F. the rectal rise per hour is 4° F.

If the hot air be moving, much greater tolerance of the heat of it is exhibited by human subjects. Thus, in an air-current moving one hundred and seventy linear feet per minute a wet-bulb temperature of 93° F. led to no abnormal rise of temperature.

In still and saturated air the limit of tolerance is largely reduced if muscular exertion is undertaken. Experiment showed that leisurely climbing at the rate of thirteen feet per minute reduced the maximum limit of tolerance by 10° F.

The cardinal symptoms accompanying this abnormal rise of body-heat were these:—

1. Increase of pulse-rate, and throbbing in the head.
2. Dyspnœa, especially on exertion.
3. Exhaustion.

The increase in the rate of the pulse was about twenty beats per minute for each degree Fahrenheit of advance in rectal temperature. Hyperpnœa did not become noticeable until the rectal temperature exceeded 102° F.

The practical conclusion is that in motionless and warm air, what really matters to persons present in it is neither the temperature of the air, nor its relative saturation, nor the absolute percentage of aqueous vapour it contains, but the temperature shown by the wet-bulb thermometer. If this exceeds about 78° F. continuous hard work becomes impossible, while if it exceeds 88° F. ordinary people cannot even stay in such air for any considerable length of time.

